# Copper radionuclides for theranostic applications: towards standardisation of their nuclear data. A mini-review

**DOI:** 10.3389/fchem.2023.1270351

**Published:** 2023-09-29

**Authors:** Mazhar Hussain, Syed M. Qaim, Ingo Spahn, M. Naveed Aslam, Bernd Neumaier

**Affiliations:** ^1^ Institut für Neurowissenschaften und Medizin, INM-5: Nuklearchemie, Forschungszentrum Jülich (FZJ), Jülich, Germany; ^2^ Department of Physics, Government College University Lahore (GCUL), Lahore, Pakistan; ^3^ Department of Physics, COMSATS University Islamabad, Lahore, Pakistan

**Keywords:** radionuclides of copper, theranostic approach, nuclear reaction cross section, nuclear model calculation, standardisation of nuclear data, thick target yield

## Abstract

Copper has several clinically relevant radioisotopes and versatile coordination chemistry, allowing attachment of its radionuclides to biological molecules. This characteristic makes it suitable for applications in molecular imaging or radionuclide targeted therapy. Of particular interest in nuclear medicine today is the theranostic approach. This brief review considers five radionuclides of copper. These are Cu-60, Cu-61, Cu-62, Cu-64, and Cu-67. The first four are positron emitters for imaging, and the last one Cu-67 is a β^–^-emitting radionuclide suitable for targeted therapy. The emphasis here is on theory-aided evaluation of available experimental data with a view to establishing standardised cross-section database for production of the relevant radionuclide in high purity. Evaluated cross section data of the positron emitters have been already extensively reported; so here they are only briefly reviewed. More attention is given to the data of the ^68^Zn(p,2p)^67^Cu intermediate energy reaction which is rather commonly used for production of ^67^Cu.

## 1 Introduction

Copper is an essential trace element in all living systems. It has versatile coordination chemistry (Blower et al., 1996; Wadas et al., 2007) which allows its metallation with various chelators, such as DOTA (1,4,7,10-tetraazacyclododecane tetraacetic acid), NOTA (1,4,7-triazacyclononane triacetic acid) etc., that can be conjugated to biological targeting molecules like peptides, proteins and antibodies (Blower et al., 1996; Wadas et al., 2007; Aluicio-Sarduy et al., 2018). Thus suitable radionuclides of copper attached to those molecules have great potential in molecular imaging and/or radionuclide targeted therapy, i.e., in following the theranostic approach, which entails the use of two radionuclides of the same element in identical chemical form, one a positron emitter for measuring the distribution kinetics of the radioactivity in the body via Positron Emission Tomography (PET), and the other a radionuclide emitting corpuscular radiation (β^–^, α or Auger electrons) useful for internal radiotherapy. The two nuclides are denoted as “matched pair” (Herzog et al., 1993; Rösch et al., 2017; Qaim et al., 2018). The radionuclides of copper of theranostic interest are listed in Table 1. They consist of four positron-emitting radionuclides, namely, ^60^Cu(T_1/2_ = 23.7 min), ^61^Cu(T_1/2_ = 3.33 h), ^62^Cu(T_1/2_ = 9.67 min) and ^64^Cu(T_1/2_ = 12.7 h), and the β^–^emitting radionuclide ^67^Cu (T_1/2_ = 61.83 h). Whereas ^60^Cu and ^62^Cu, being rather short-lived, have found only limited application in PET measurements (Wallhaus et al., 1998; McCarthy et al., 1999; Ng et al., 2014), ^61^Cu and ^64^Cu are more widely used. In particular ^64^Cu is gaining increasing significance due to its almost ideal decay characteristics for PET imaging (Williams et al., 2005). The counterpart radionuclide ^67^Cu is of considerable interest in internal radiotherapy because of its suitable half-life and β^–^energy. The “matched pairs” ^61^Cu/^67^Cu and ^64^Cu/^67^Cu thus constitute very important theranostic pairs. Furthermore, because of its β^–^and Auger electron emission component, the radionuclide ^64^Cu is also of interest in radionuclide targeted therapy.

**TABLE 1:** Standardised decay and production data of some copper radionuclides of theranostic interest.

Radionuclide	Decay data				Production data		
	T_½_	Mode of decay (%)	Maximum β particle energy (keV)	E_γ_ in keV(%)	Nuclear reaction	Optimum energy range (MeV)	Calculated yield (MBq/μAh) [Ref.]
^60^Cu	23.7 min	β^+^ (92)	2,500	826.4 (21.7)	^60^Ni(p,n)	15→7	3,400 [our value]^b^
		EC (8)		1,332.5 (88.0)			
				1791.6 (45.4)			
^61^Cu	3.34 h	β^+^ (61)	1,300	282.9 (12.2)	^61^Ni(p,n)	15→7	1,418 [our value]^c^
							1,434 [CRP]^d^
		EC (39)		656.0 (10.4)	^64^Zn(p,α)	18→11	288 [our value]^e^
							257 [CRP]^d^
^62^Cu	9.67 min	β^+^ (98)	2,935	875 (0.15)	^63^Cu(p,2n)^62^Zn→^62^Cu	30→14	233 [CRP]^f^
		EC (2)		1,173 (0.34)	^62^Ni(p,n)	15→7	45,000[our value]^c^
							38,240 [CRP]^d^
^64^Cu	12.7 h	EC (43.8)		1,345.7(0.47)^a^	^64^Ni(p,n)	12→8	304 [our value]^g^
		β^+^ (17.8)	653				306 [CRP]^d^
		β^–^ (38.4)	571				
^67^Cu	61.8 h	β^–^ (100)	577	184.6 (48.6)	^70^Zn(p,α)	25→10	4.4 [CRP]^h^
					^68^Zn(p,2p)	80→30	42 [CRP]^h^
							38 [CRP]^i^

^a^ There is discrepancy between this value and our reported value of 0.54% (Qaim et al., 2007).

^b^ Not standardised. Yield calculated from theory-validated experimental excitation function (Uddin et al., 2016).

^c^ From our evaluated data (Aslam and Qaim, 2014a).

^d^ From IAEA-CRP, evaluated data (Tárkányi et al., 2019).

^e^ From our evaluated data (Aslam and Qaim, 2014b).

^f^ From IAEA-CRP, evaluated data (Hermanne et al., 2018).

^g^ From our evaluated data (Aslam et al., 2009).

^h^ From IAEA-CRP, evaluated data (Qaim et al., 2011).

^i^ From IAEA-CRP, evaluated data (Tárkányi et al., 2022).

The most significant decay data and the important production methods of the five radionuclides under consideration are also given in Table 1. Both decay data and production methodologies have been amply described (Qaim et al., 2018; Qaim, 2019; NUDAT 3.0, 2023; Nichols, 2022; Qaim, 2017; Qaim et al., 2019). In this brief review therefore we concentrate only on a special aspect, namely, the standardisation (also called “evaluation”) of nuclear data of those five radionuclides. As far as we know, to date such a review has not been written.

## 2 Standardised decay data: discrepancies and deficiencies

The decay data have conventionally received more attention with regard to their standardisation, and detailed evaluated mass decay chains are available for the above-mentioned five radionuclides (Junde et al., 2005; Nichols et al., 2012; Browne and Tuli, 2013; Zuber and Singh, 2015; Singh and Chen, 2021). The data given in Table 1 are all standardised, especially the half-lives, the γ-ray energies and their intensities, as well as the β^+^(β^–^) energies and their intensities. They were taken from NUDAT which is based on evaluated mass decay chains. Yet some uncertainties do exist. The intensity of the weak 1,345.7 keV γ-ray of ^64^Cu, for example, is slightly controversial. The reported standardised value is (0.47 ± 0.01) % (Bé et al., 2012). A later measurement using ^64^Cu in a solution volume of 5 mL gave the same value for the intensity of this γ-ray (Pibida et al., 2017). But an independent experiment done earlier at FZJ (Qaim et al., 2007), utilizing a cyclotron-produced, radiochemically separated, highly pure thin point source gave a value of (0.54 ± 0.03) %. Incidentally, in that work the intensities obtained for β^+^, β^–^and EC emissions were exactly the same as the standardised values. The discrepancy is thus specific to the determination of the 1,345.7 keV γ-ray. An independent experiment using a properly prepared thin sample of ^64^Cu is therefore suggested to solve the discrepancy.

An important consideration in quantification of a PET measurement is the intensity of the positrons emitted from the radionuclide (I_β+_). In most cases this intensity (% of decay) is derived from a balance of various γ-transitions described in the decay scheme. But often there are uncertainties in the reported values. A direct experimental determination of the positron emission intensity involving a spectrometric analysis of the 511 keV annihilation radiation and K_α_ X-rays, as developed at FZJ, appears to provide more accurate results (Qaim et al., 2007). The I_β+_ value for ^64^Cu obtained this way amounted to 17.8% and is now regarded as the standard value. For ^60^Cu, ^61^Cu, and ^62^Cu, so far such a direct measurement has not been performed. On the other hand, the I_β+_ values for ^60^Cu and ^62^Cu, being 93% and ∼100%, respectively, are rather strong; the uncertainty is therefore assumed to be small. Furthermore, due to very limited use of those radionuclides, the reported I_β+_ values appear to be satisfactory. Regarding the radionuclide ^61^Cu, on the other hand, the reported I_β+_ value of 61% is rather uncertain (Qaim, 2017; Qaim et al., 2019). In view of somewhat enhancing interest in this radionuclide, a direct measurement of the I_β+_ value, as done for ^64^Cu, appears to be of some urgency.

As far as the decay data of ^67^Cu are concerned, there appears to be no discrepancy (Junde et al., 2005) and the energies and intensities of all emitted radiations given in Table 1 may be regarded as standardised values.

## 3 Methodologies for standardisation of production data

### 3.1 General development

In contrast to neutron-induced reactions, the standardisation of charged-particle induced reaction cross sections, needed for production of radionuclides at cyclotrons/accelerators, remained initially somewhat neglected (Qaim, 2020). From 1995 onwards, however, the IAEA got interested in this field, and the relevant data compilation and evaluation efforts were intensified. The former led to improvement of the international EXFOR file and the standardisation of those data was initiated and followed through three successive Coordinated Research Projects (CRPs). Since no evaluation methodology for charged-particle data existed in the beginning, in the first CRP related to commonly used diagnostic radionuclides, the work was rather empirical and reliance was placed on statistical fitting of concordant set of data (Gul et al., 2001). In the second CRP dealing with therapeutic radionuclides, theory was also introduced to some extent. Calculations were done using the codes ALICE-IPPE and EMPIRE for comparison with the experimental data (Qaim et al., 2011). The third and last CRP in the series became a very extensive endeavour dealing with a large number of novel radionuclides (Tárkányi et al., 2019). However, the selection/rejection of the experimental data in the evaluation process remained rather empirical, and standardised curves were obtained by statistical fittings. For comparison, only the results of the global theoretical file TENDL were considered. All data for charged-particle production of medical radionuclides evaluated under those CRPs are available on the website of the IAEA [Medical Portal (iaea.org)].

### 3.2 Theory-assisted selection of data in the standardisation process

Standardisation work on charged-particle induced reaction cross sections has also been done outside the IAEA-CRPs mentioned above. In those studies extensive use was made of nuclear model calculations to ascertain the most concordant set of data for a given nuclear reaction, followed by polynomial fitting of the selected data. Based on a suggestion made by Sudár et al. (2002), the standardisation methodology was extensively developed under a German-Pakistan cooperation (Hussain et al., 2009, 2010; Aslam and Qaim, 2014a; Aslam and Qaim, 2014b; Amjed et al., 2016, 2020; Ali et al., 2019). It consists of a comparison of the experimental data with the results of a nuclear model calculation, whereby the input parameters are adjusted within their recommended limits (RIPL-3). The basic relation for obtaining the evaluated cross section is developed as

$$\sigma_{\mathrm{ev}}\left(E\right)=f\;\left(E\right)\;\sigma_{\mathrm{model}}\;\left(E\right)$$

where *σ*_*ev*_ *(E), σ*_*model*_ *(E) and f (E)* are the evaluated cross section, model calculated cross section and the energy-dependent normalisation factor, respectively. The ratio of experimental to calculated data is plotted as a function of energy, followed by a polynomial fitting to estimate the f (E). The procedure is repeated with all model calculations. The recommended data are generated by averaging the normalised model calculations.

For nuclear model calculations, in our collaboration four major codes, namely, STAPRE, ALICE-IPPE, EMPIRE and TALYS (Koning et al. 2005) were used. With the exception of ALICE-IPPE, which is based purely on the exciton precompound model, the other codes entail a combination of compound and precompound processes, with some consideration of direct interactions. They reproduce the experimental data with varying degree of success. In each calculation therefore, the optical model parameters were varied within their recommended limits to obtain a fit as close as possible to the experimental data. The recommended data for the particular production reaction were then generated as outlined earlier. For uncertainty estimates, a confidence limit of 95% was adopted. With constant improvements in the code TALYS in recent years (Koning et al. 2023), its use has become more universal.

## 4 Standardised (recommended) cross-section data

For all 5 radionuclides under consideration, several production routes were studied (see below). However, in Table 1 we give for each radionuclide only the more commonly used production reactions, the optimum energy ranges derived and the thick target yields calculated from the standardised excitation functions. Each radionuclide is discussed below individually.

### 4.1 Positron emitters

#### 4.1.1 ^60^Cu

The most suitable production route for this radionuclide is the ^60^Ni(p,n)^60^Cu process. However, no attempt has been made to standardise its cross-section data either in the IAEA-CRP or by us. On the other hand, a detailed experimental and theoretical study of the excitation function of this reaction has been carried out by Uddin et al. (2016). We used those data as reference values to deduce the optimum energy range (E_p_ = 15→7 MeV) for the production of ^60^Cu, and also calculated its expected thick target yield. This process has been developed to produce high-purity ^60^Cu on a clinical scale (McCarthy et al., 1999).

#### 4.1.2 ^61^Cu

The cross-section data for the reactions ^61^Ni(p,n)^61^Cu, ^62^Ni(p,2n)^61^Cu, ^60^Ni(d,n)^61^Cu and ^58^Ni(α,p)^61^Cu were standardised by Aslam and Qaim (2014a) and those for the reactions ^64^Zn(p,α)^61^Cu, ^64^Zn(d,αn)^61^Cu, ^59^Co(^3^He,n)^61^Cu and ^59^Co(α,2n)^61^Cu by Aslam and Qaim (2014b). The standardised numerical data are given in those two publications. The ^59^Co(α,2n)^61^Cu reaction can be used if an α-particle beam of about 40 MeV is available (Fukumura et al., 2004). However, the reactions ^61^Ni(p,n)^61^Cu and ^64^Zn(p,α)^61^Cu have been more commonly used for production. The suitable energy ranges for those two reactions were deduced. The calculated yields of ^61^Cu from standardised curves by us (Aslam and Qaim, 2014a; Aslam and Qaim, 2014b) and those by the IAEA-CRP (Tárkányi et al., 2019) are given in Table 1. They agree within about 1% for the ^61^Ni(p,n)-reaction and about 10% for the ^64^Zn(p,α)-reaction. For both reactions, using highly-enriched targets, clinical scale production of high-purity ^61^Cu has been reported (McCarthy et al., 1999; Thieme et al., 2013).

#### 4.1.3 ^62^Cu

This short-lived positron emitting radionuclide is obtained via two routes:

a) ^nat^Cu(p,xn)^62^Zn→^62^Cu (generator)
b) ^62^Ni(p,n)^62^Cu

The data for the reaction ^nat^Cu(p,xn)^62^Zn have been very well standardised because it is an important monitor reaction (IAEA report, Gul et al., 2001) and the calculated yield of ^62^Zn over the suitable energy range (E_p_ = 30→14 MeV) is given in Table 1. Use of an enriched target is not necessary. This route is the method of choice for the production of ^62^Cu (Wallhaus et al., 1998; Ng et al., 2014). The data for the reaction ^62^Ni(p,n)^62^Cu were standardised by us (Aslam and Qaim, 2014a) and the IAEA-CRP (Tárkányi et al., 2019). The yields agree within about 15%. This route gives a very high yield of the product but, in order to achieve high radionuclidic purity, an enriched target is needed. It has seldom been used for production.

#### 4.1.4 ^64^Cu

Cross-section data of a large number of reactions leading to the formation of this radionuclide have been standardised both by us (Aslam et al., 2009) and under an IAEA-CRP (Qaim et al., 2011). They include the reactions ^64^Ni(p,n)^64^Cu, ^64^Ni(d,2n)^64^Cu, ^68^Zn(p,αn)^64^Cu, ^66^Zn(p,2pn)^64^Cu, ^64^Zn(d,2p)^64^Cu, ^66^Zn(d,α)^64^Cu, ^nat^Zn(d,x)^64^Cu and a few others. Out of all those reactions, however, the ^64^Ni(p,n)^64^Cu process on highly-enriched target is the most interesting. Its thick target yields reported by us and the IAEA-CRP agree within 1%. Initially proposed by the Jülich group (Szelecsényi et al., 1993) and further developed by the St. Louis group (McCarthy et al., 1997), the technology was improved over the years (for a review cf. Qaim et al., 2018), and today this reaction has become the method of choice for large scale production of high-purity and high-specific-activity ^64^Cu.

### 4.2 Therapeutic radionuclide ^*67*^ *Cu*

This β^–^-emitting therapeutic radionuclide has been of interest for more than 40 years and its production methods have been reviewed by several groups (Qaim et al., 2011; Qaim, 2012; IAEA-report; Smith et al., 2012; Qaim et al., 2018; Mou et al., 2022). Considerable industrial efforts are underway to produce it via the ^68^Zn(γ,p)^67^Cu process. Its excitation function is known fairly well. We concentrated on four charged-particle induced reactions. Three of them have been investigated in the low-energy range. They are ^70^Zn(p,α)^67^Cu (Levkovskii, 1991; Kastleiner et al., 1999; Dellepiane et al., 2023), ^70^Zn(d,αn)^67^Cu (Kozempel et al., 2012; Nigron et al., 2021) and ^64^Ni(α,p)^67^Cu (Skakun and Qaim, 2004; Ohya et al., 2018; Uddin et al., 2018; Takács et al., 2020). An evaluation of the cross section data was, however, carried out only for the ^70^Zn(p,α)^67^Cu reaction under an IAEA-CRP (Qaim et al., 2011). The new data by Delllepiane et al. (2023) up to 18 MeV fit well in the evaluated curve. Thus a reliable standardised database is available for this reaction up to 30 MeV. This method has been used for ^67^Cu production in MBq quantities at 24–30 MeV cyclotrons (Hilgers et al., 2003; Lee et al., 2022). Very recently some new data have been reported for the ^70^Zn(p,x)^67^Cu process up to proton energy of about 70 MeV (Pupillo et al., 2020). The cross section increases sharply beyond 40 MeV. An evaluation of the data would be meaningful when more information is available. The fourth reaction, namely, ^68^Zn(p,2p)^67^Cu, is presently often used for production purposes. The standardisation of production cross sections was attempted under an IAEA-CRP (Qaim et al., 2011). Another IAEA-CRP version has also been presented (Tárkányi et al., 2022). Jung et al. (2023) reported extensive new cross-section measurements in the higher energy range. We discuss critically all reported data.

**FIGURE 1:**
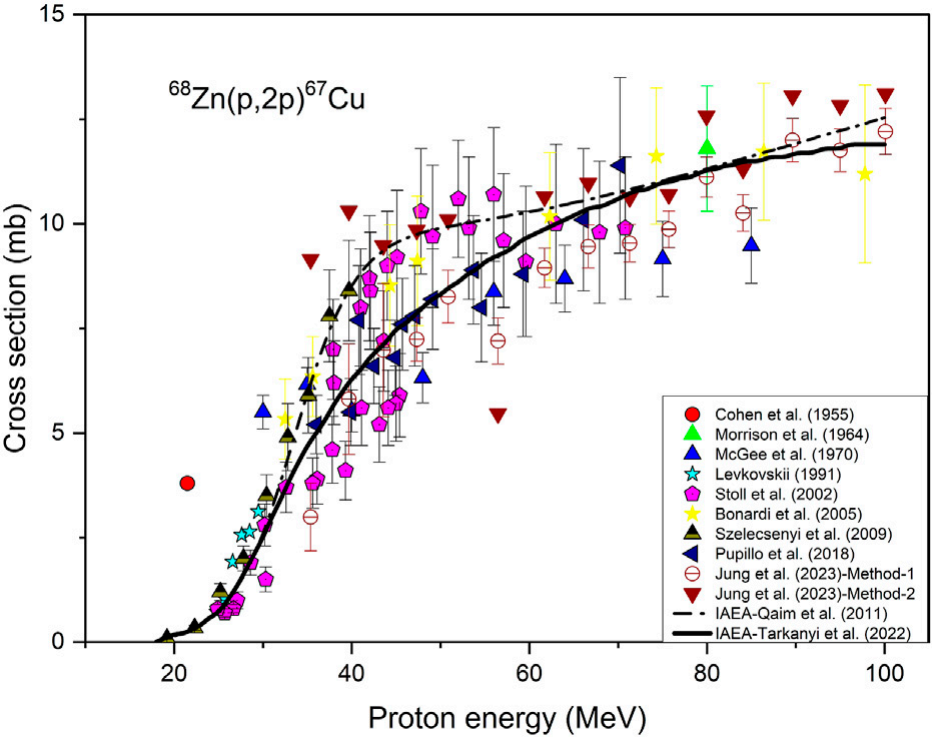
Normalised experimental data and IAEA-evaluated reaction cross section curves for the ^68^Zn(p,2p)^67^Cu process.

For this reaction, twelve experiments have been reported in the EXFOR library of the IAEA (http://www-nds.iaea.org/exfor/exfor.htm) over the proton energy range up to 430 MeV. For evaluation, however, the data only up to 100 MeV are interesting, i.e., leaving out some data points in the higher energy region (Morrison et al., 1962; Morrison et al., 1964; Mirzadeh et al., 1986). Cohen et al. (1955) measured only one cross section value at 21.5 MeV which was found to be very discrepant. McGee et al. (1970) performed measurements at eight energies covering the range from 30 to 85 MeV. They used the reference of Meadows (1953) for the monitor reaction. The present status of that monitor reaction led us to correct those data. Similarly the Levkovskii data (1991) were reduced by 25% because of the use of wrong monitor cross section (Qaim et al., 2014). Stoll et al. (2002) performed the experiment over a wide energy range of 24.9–70.8 MeV at two cyclotrons (JULIC and PSI accelerator) using enriched ^68^Zn (98.3%) thin target samples and radiochemical separation of ^67^Cu. The experimental data were generally consistent but a few data points showed a systematic lower trend in the energy range of 35–45 MeV investigated at the cyclotron JULIC. Bonardi et al. (2005) studied this reaction up to 141 MeV using thin target foils of Zn. The data were consistent but with large uncertainties in all energy regions. Szelecsényi et al. (2009) reported the data for this reaction up to 40 MeV. They used enriched ^68^Zn (≥99%) as the target material. Recently two detailed measurements have been reported for this reaction using enriched ^68^Zn targets, one by Pupillo et al. (2018) and the other by Jung et al. (2023). Pupillo et al. (2018) measured the cross sections after radiochemical separation while Jung et al. (2023) used two analytical methods without radiochemical separation. Both datasets were found to be consistent. All the normalised data are plotted in Figure 1 as a function of proton energy. The IAEA evaluated data curves (Medical Portal (iaea.org)) are also shown in Figure 1. It is evident that those two evaluations are not fully supported by the new data. In particular the updated curve (Tárkányi et al., 2022) appears to be too low. Considering all the data published we conclude that a new evaluation is necessary using the theory-assisted selection of data. For practical production of ^67^Cu via this route, however, the energy range E_p_ = 80→30 MeV remains the most suitable (Qaim, 2012). This method has been practically used in clinical scale production of ^67^Cu (Katabuchi et al., 2008; Medvedev et al., 2012).

## 5 Discussion

The decay data of the 5 radionuclides of copper relevant for theranostic applications are well standardised, except for the intensity of the weak γ-line of ^64^Cu at 1,345.7 keV where some discrepancy exists. This discrepancy needs to be solved, especially because some radionuclide producers use this γ-ray for determination of the total radioactivity of ^64^Cu.

The data for production of ^61^Cu, ^62^Cu and ^64^Cu via the more common routes are well evaluated. For the standardisation of production cross sections of ^60^Cu, however, more measurements are needed. With regard to the data for the production of the therapeutic radionuclide ^67^Cu, presently considerable efforts are underway. Standardised data for the reaction ^70^Zn(p,x)^67^Cu are available up to 30 MeV which consists of the (p,α) reaction. In the higher energy region up to 70 MeV, however, the cross section increases rapidly, possibly due to the onset of the many nucleon emission processes like ^70^Zn(p,2p2n)^67^Cu. But more measurements and a critical evaluation are needed to obtain standardised data for this process. The presently rather commonly used intermediate reaction ^68^Zn(p,2p)^67^Cu was evaluated under two IAEA-CRPs. The newest measurements, however, show some deviations from the evaluated data. A new critical evaluation should thus be very meaningful. In order to estimate the specific activity of the radionuclide produced, it is also imperative to determine via model calculation the inactive material, i.e., ^65^Cu, co-produced with the respective radionuclide.
